# Gene therapy for cystic fibrosis: Challenges and prospects

**DOI:** 10.3389/fphar.2022.1015926

**Published:** 2022-10-11

**Authors:** Hongshu Sui, Xinghua Xu, Yanping Su, Zhaoqing Gong, Minhua Yao, Xiaocui Liu, Ting Zhang, Ziyao Jiang, Tianhao Bai, Junzuo Wang, Jingjun Zhang, Changlong Xu, Mingjiu Luo

**Affiliations:** ^1^ Department of Histology and Embryology, School of Clinical and Basic Medical Sciences, Shandong First Medical University & Shandong Academy of Medical Science, Jinan, Shandong, China; ^2^ Shandong Provincial Key Laboratory of Animal Biotechnology and Disease Control and Prevention, College of Animal Science and Veterinary Medicine, Shandong Agricultural University, Tai’an, China; ^3^ The Affiliated Tai’an City Central Hospital of Qingdao University, Tai’an, Shandong, China; ^4^ Department of Neurology, The Second Affiliated Hospital of Shandong First Medical University, Tai’an, Shandong, China; ^5^ The Reproductive Medical Center of Nanning Second People’s Hospital, Nanning, China; ^6^ National Center for International Research of Bio-targeting Theranostics, Guangxi Key Laboratory of Bio-targeting Theranostics, Collaborative Innovation Center for Targeting Tumor Diagnosis and Therapy, Guangxi Talent Highland of Bio-targeting Theranostics, Guangxi Medical University, Nanning, China

**Keywords:** cystic fibrosis, gene therapy, viral vectors, animal models, airway delivery

## Abstract

Cystic fibrosis (CF) is a life-threatening autosomal-recessive disease caused by mutations in a single gene encoding cystic fibrosis transmembrane conductance regulator (CFTR). CF effects multiple organs, and lung disease is the primary cause of mortality. The median age at death from CF is in the early forties. CF was one of the first diseases to be considered for gene therapy, and efforts focused on treating CF lung disease began shortly after the CFTR gene was identified in 1989. However, despite the quickly established proof-of-concept for *CFTR* gene transfer *in vitro* and in clinical trials in 1990s, to date, 36 CF gene therapy clinical trials involving ∼600 patients with CF have yet to achieve their desired outcomes. The long journey to pursue gene therapy as a cure for CF encountered more difficulties than originally anticipated, but immense progress has been made in the past decade in the developments of next generation airway transduction viral vectors and CF animal models that reproduced human CF disease phenotypes. In this review, we look back at the history for the lessons learned from previous clinical trials and summarize the recent advances in the research for CF gene therapy, including the emerging CRISPR-based gene editing strategies. We also discuss the airway transduction vectors, large animal CF models, the complexity of CF pathogenesis and heterogeneity of CFTR expression in airway epithelium, which are the major challenges to the implementation of a successful CF gene therapy, and highlight the future opportunities and prospects.

## 1 Introduction

**FIGURE 1:**
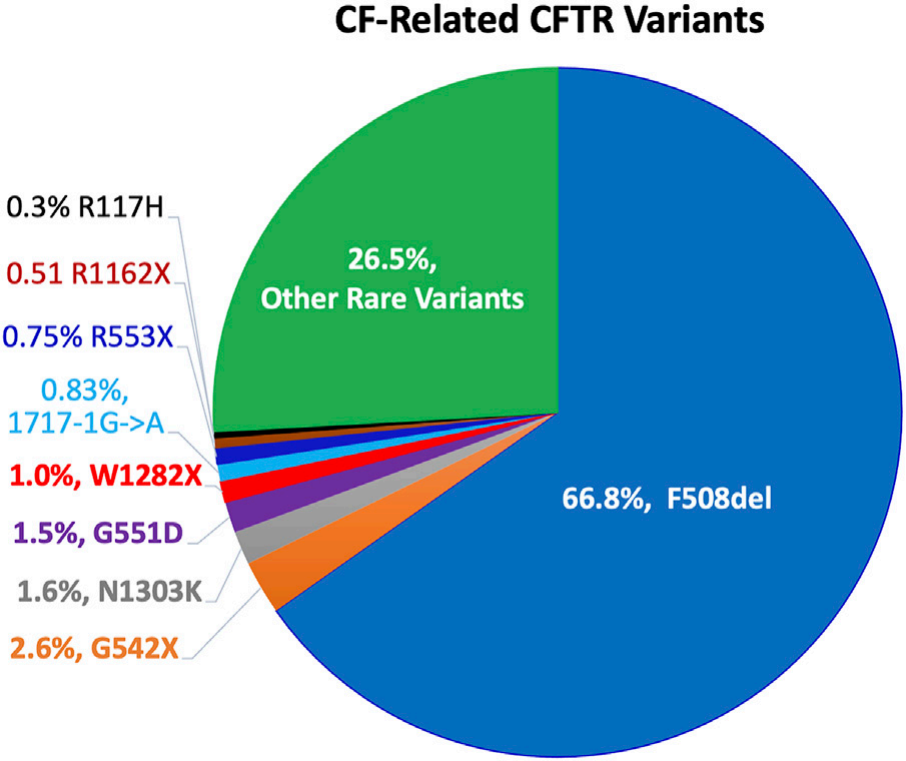
Worldwide distribution of disease-causing CFTR variants. More than 2,000 different mutations in *CFTR* have been identified, and ∼400 mutations are confirmed to be disease-causing. (http://www.genet.sickkids.on.ca/app). Pie chart represents the frequencies of common CF mutations (>0.3%) in population worldwide. Notably, the distribution and frequency of CFTR variants vary in different countries and ethnic groups.

Cystic fibrosis (CF) is an autosomal-recessive disease affecting over 80,000 people worldwide (Brown et al., 2017; Scotet et al., 2020a). CF is caused by mutations in a single gene encoding a product named cystic fibrosis transmembrane conductance regulator (CFTR) (Riordan et al., 1989; Rommens et al., 1989). CFTR is as a cyclic adenosine monophosphate (cAMP)-dependent ion channel protein that conducts chloride (Cl^−^) and bicarbonate (HCO_3_^−^) ions across the epithelia, and it plays an important role in epithelial ion transport and fluid homeostasis (Welsh and Smith, 1993; Zabner et al., 1998; Gadsby et al., 2006; Pezzulo et al., 2012; Stoltz et al., 2015; Kunzelmann et al., 2017). Mutations in CFTR can result in reduced or absent expression, or malfunction, leading to *CF.* Nearly 400 of the ∼2,000 identified *CFTR* mutations have been confirmed to be disease-causing mutations (http://www.genet.sickkids.on.ca/app). The classification system of the Cystic Fibrosis Foundation (www.cff.org) groups these mutations into five classes by the problems in: 1) protein production (no synthesis), 2) defective protein processing, 3) defective gating, 4) low conductance, and 5) insufficient quantities of protein expression (low synthesis or increased turnover). Worldwide, the distribution and frequency of CFTR variants vary in different countries and ethnic groups, but only a limited number of mutant alleles display a frequency higher than 1% (Ferec and Cutting, 2012). An early study conducted in United States found that CF more frequently occurred in non-Hispanic Caucasian (1/2,500) and Ashkenazi Jews (1/2,300), compared to that in Hispanic Caucasian (1:13,500), African Americans (1:15,100), and Asian Americans (1:35,100) (Palomaki et al., 2004). Figure 1 presents the common CF-related *CFTR* mutations of >1% frequency in population worldwide. The most common CF-associated mutation is F508del, an example of a Class 2 processing-defect mutation. F508del accounts for approximately two-thirds of CF alleles (66.8%) (Bobadilla et al., 2002). The other common mutations include G542X (2.6%, Class 1), N1303K (1.6%, Class 2), G551D (1.5%, Class 3), W1282X (1.0%, Class 1) (Zvereff et al., 2014; Chamayou et al., 2020; Scotet et al., 2020a; Scotet et al., 2020b; Petrova et al., 2021; Yiallouros et al., 2021).

CF affects various organ systems and is associated with dehydration of the epithelial surface, reduction in the pH of secretions, and excessive mucus production and obstruction (Shteinberg et al., 2021). Lung disease is the primary cause of CF morbidity and mortality (Stoltz et al., 2015). Patients with CF suffer progressive damage in the lung from recurrent respiratory infections and exaggerated inflammatory response, eventually, respiratory failure. In the past, therapies for patients with CF aimed to provide symptomatic care. With improved treatments and better healthcare, now the median age of death from CF is in the early forties (Keogh et al., 2018). Recent advances in pharmaceutical rescue of mutant CFTR function using CFTR modulators have made it possible to target the underlying genetic cause of CF and further increase the quality of life for patients. CFTR modulators are small molecule drugs that were identified from high throughput screening for correcting the impaired CFTR trafficking to the plasmid membrane (corrector) or for increasing the CFTR channel conductance (potentiator) (Lopes-Pacheco, 2019). Thus, CFTR modulator therapies are genotype-dependent, effective on patients who are with the CFTR mutants affecting protein trafficking (Class II), gating and conductance (Class III and IV). To date, four CFTR modulator therapies have been approved and ∼90% CF patients with certain CFTR mutants are benefited with therapeutic outcomes (Ramsey et al., 2011; Rowe et al., 2017; Keating et al., 2018; Taylor-Cousar et al., 2018; Heijerman et al., 2019; Middleton et al., 2019). While the potentiator Ivacaftor is eligible for patients with the G551D mutation (Ramsey et al., 2011), Trikafta, the combination of three correctors and potentiator, has been used to treat the patients carrying one copy of the most common F508del mutant (Middleton et al., 2019). However, CFTR modulator therapies require life-long drug administration, their long-term potential side effects remain unclear (Lopes-Pacheco, 2019). Currently, approximately 10% of patients with CF, who have drug-refractory missense mutations, who produce little to no CFTR protein, or who cannot tolerate CFTR modulators, are without an option of CFTR modulator therapy and still rely on symptomatic treatment (Clancy et al., 2019; Middleton et al., 2019). For treatments to the patients left behind, the interest of pharmaceutical industry to explore novel CF modulators to target those rare CFTR mutations is still growing (Fajac and Sermet, 2021; Laselva et al., 2021). The RNA-based techniques such as engineered tRNA (Lueck et al., 2019) or antisense oligonucleotides (ASOs) (Michaels et al., 2020) to mask the nonsense mutation or to mediate splicing modulation are potentially useful as therapeutic approaches. Although the emerging mRNA delivery technique is thought to be feasible to treating any CF patients, independent of underlying mutations, using the mRNA encoding CFTR (Haque et al., 2018; Robinson et al., 2018), this treatment necessitates life-time administration, similarly as do the CFTR modulator therapies, engineered tRNA or ASOs. In contrast, gene therapy, either *via* gene addition or gene editing, is able to alter the CFTR expression at DNA level, and such alternation is expected to be persistent in whole life of the recipient cells. As the mutation-agnostic, gene therapy is suitable for all patients with CF, regardless of their genotype (Griesenbach et al., 2016). Thus, gene therapy for CF lung disease remains an attractive approach for treating all the population with CF, not only for other currently lacked of the option of CFTR modulator therapy (Choi and Engelhardt, 2021; Mercier et al., 2021). The goal for gene therapy as a cure for CF lung disease has been pursuing for almost 30 years, desirable outcome has not yet met. Researchers in both industry and academic have contributed substantial effort and talents to tackle the challenges encountered during this long journey. New animal models and more efficacious gene transfer agents have been developed to move the field in the path to success. Several recent excellent review articles have summarized and discussed the lessons and achievements from the last 3 decades (Cooney et al., 2018a; Donnelley and Parsons, 2018; Yan et al., 2019; Tang et al., 2020a; Allan et al., 2021; Lee et al., 2021). In this review, after a brief description of the lessons learned from the previous unsuccessful clinical trials of CF gene therapy in an alignment with recent progress in animal models and viral vectors as tools of gene delivery, we will focus on the major challenges to the implementation of the gene therapy for CF lung disease. We will discuss why such difficulties are hindering the advance of *CFTR* gene addition therapies toward a successful clinical trial and how researchers are tackling these problems now. We also discuss the novel non-viral vector delivery strategies, and the potential and limitation of the CRISPR-based gene editing as a path toward a cure for *CF.*

## 2 History of gene therapy for CF lung disease

CF was first described as a specific disease in 1938 (Andersen, 1938), and the *CFTR* gene was discovered in 1989, with multiple mutations identified responsible for CF, including the most common F508del (Riordan et al., 1989; Rommens et al., 1989). Shortly after the cloning of *CFTR*, the goal to develop gene therapy as a cure for CF lung disease was eagerly pursued by both academia and industry (Flotte, 1993; Cooney et al., 2018a). In 1993, a pioneer study intended to compensate for defective *CFTR* in the nasal airway *via* gene replacement using an E1-deleted recombinant adenoviral (rAd) vector to deliver a normal copy of *CFTR* complementary DNA (cDNA) to the nasal epithelium of three patients with *CF.* Albeit not placebo-controlled, this study demonstrated proof-of-concept for gene therapy; specifically, for the transient correction of Cl^−^ transport after vector inoculation (Zabner et al., 1993). Since then, nine further rAd vector trials to treat CF lung disease were conducted, but it turned out that the first generation of rAd vectors were not suitable for gene therapy because of transient gene expression and the strong immunogenicity (Joseph et al., 2001; Perricone et al., 2001).

Recombinant adeno-associated virus (rAAV) vectors are the most promising vectors for human gene therapy. Several rAAV-based gene therapies have been approved for clinical treatments (Kuzmin et al., 2021). CF lung disease gene therapy using rAAV-2 vector was evaluated in clinics from 1998 to 2007, led by the Targeted Genetics Corporation (Guggino and Cebotaru, 2017, 2020). Phase I single-dose studies demonstrated the safety of the gene transfer agent, tgAAVCF, in human application and its successful delivery to the maxillary sinuses and lungs of patients with *CF.* Although vector-derived *CFTR* mRNA expression was beyond the detection limit, the sinus transepithelial potential was assessable on days 7 and 14 after infection, indicating the transient functional restoration (Wagner et al., 1998; Aitken et al., 2001; Flotte et al., 2003). Phase II trials of two doses of vectors with an interval of 30 days found that 25% of the patients treated with tgAAVCF demonstrated an improvement of >10% in forced expiratory volume in 1 s (FEV1) while no improvement was observed in the placebo group (Wagner et al., 2002; Moss et al., 2004; Moss et al., 2007). Despite these promising results, the overall outcomes did not meet the primary efficacy endpoint of an improvement in lung function.

Non-viral vectors are advantageous because they are less immunogenic than viral vectors for repeat dosing (Yin et al., 2014). Studies on non-viral *CFTR* delivery revealed that the cationic liposome (GL67A)-formulated plasmid-based *CFTR* gene transfer to the airway epithelia (nose and lung) was safe (Alton et al., 1999) and also feasible for repeat dosing (Hyde et al., 2000). Using the most potent non-viral vector (GL67A complexed with pGM169, a CpG nucleotide-free CTTR expression plasmid) (Hyde et al., 2008; Alton et al., 2013; Alton et al., 2014), the United Kingdom Cystic Fibrosis Gene Therapy Consortium (UK CFGTC) conducted a double-blinded, placebo-controlled, multidose trial to assess its clinical benefit. This is to date the largest CF gene therapy phase IIb trial with 116 patients (62 active, 54 placebo). In 2015, the results of the trials were published: patients with CF who received nebulized vectors at 28-day intervals for 12 months demonstrated an increase in FEV1 of 3.7% (0.07%–7.25%) (Alton et al., 2015). However, such improvement was still insufficient to restore function in CF lungs.

Collectively, the goal of gene therapy as a cure for CF lung disease has been pursued for almost 3 decades. Selected clinical trials of the gene therapy for CF lung disease are listed in Table 1. Although some promising results have been obtained from 36 gene therapy clinical trials involving ∼600 patients with CF (Ginn et al., 2018), desirable outcomes are yet to be demonstrated. Nevertheless, these early attempts established the proof-of-concept for CF gene therapy through gene replacement, and it has taught the field many important lessons, which are discussed in the next section.

**TABLE 1:** Selected clinical trials of gene therapy for cystic fibrosis lung disease.

Start year	Registration # trial phase	Brief title	Gene transfer agent	Location	Enrolled patients	References
1993	NCT00004779 Phase 1	Phase I Pilot Study of Ad5-CB-CFTR in CF Patients	cDNA, Ad5-CB-CFTR (rAd5 vector)	United States	12	Knowles et al. (1995)
1995	NCT00004287 Phase 1	Phase I Study of Adenovirus H5.001CBCFTR in CF Patients	cDNA, H5.001CBCFTR (rAd5 vector)	United States	14	Zuckerman et al. (1999)
1995	NCT00004471 Phase 1	Phase I Pilot Study of Cationic Liposome Mediated Gene Transfer in Patients with CF	cDNA, pGT-1 (Liposome complex, DMRIE/DOPE)	United States	9	NA
1999	NCT00004533 Phase 1/2	Phase I Randomized Study of AAV-CFTR in Patients with CF	cDNA, tgAAVCF (rAAV2 vector)	United States	19	Aitken et al. (2001), Flotte et al. (2003)
2003	NCT00073463 Phase 2/3 (Terminated at phase 2B)	Safety and Efficacy of AAV-CFTR in Patients with CF	cDNA, tgAAVCF (rAAV2 vector)	United States	100	Wagner et al. (2002), Moss et al. (2004), Moss et al. (2007)
2008	NCT00789867 Phase 1/2	Single Dose of pGM169/GL67A in CF Patients	cDNA, pGM169/GL67A (Liposome complex)	United Kingdoms	35	Alton et al. (1999), Alton et al. (2013)
2012	NCT01621867 Phase 2B	Repeated Application of Gene Therapy in CF Patients	cDNA, pGM169/GL67A (Liposome complex)	United Kingdoms	130	Alton et al. (2014), Alton et al. (2015)
2018	NTC03375047 Phase 1/2 (Ongoing)	Evaluate Safety, Tolerability of Nebulized MRT5005 in Adults with CF	mRNA, MRT5005 (LNP, lipid nanoparticles)	United States	40	NA
2022 (Recruiting)	NCT05248230 Phase 1/2 (Recruiting)	4D-710 in Adult Patients with CF	cDNA (with partial deletion at R domain). 4D-710 (rAAV-4D-A101)	United States	21	NA

## 3 Lessons learned from previous trials and progress of recent research

Although the airways are non-invasively accessible, pulmonary gene transfer has proven more difficult than anticipated. The lung airways have evolved a complex array of extracellular and intracellular mechanisms to protect against pathogens and foreign invaders (Ferrari et al., 2003). Airway epithelial cells, the primary recipients of *CFTR* gene transfer, play an indispensable role as the first-line host defense in the lung and have a critical role in innate antiviral responses to infection, and thus are resistant to vector transduction or transfection (Hewitt and Lloyd, 2021). For the accession of cell receptors on the apical surface, transgene vectors must be able to penetrate the airway mucus layer (Cone, 2009) and overcome the innate defenses, e.g., mucociliary clearance (MCC), which is a self-clearing mechanism of the airways (Bustamante-Marin and Ostrowski, 2017). In airways with CF, these physical barriers are exacerbated because of the increased thickness and viscosity of the mucus and sputum (Kreda et al., 2012; Shteinberg et al., 2021). The *CFTR* transfer vectors used in previous trials have proven inefficient to transduce or transfect CF airways *via* apical luminal delivery. Researches into the transduction biology of these vectors in different airway epithelium model systems *in vitro* and in the lungs of experimental animals *in vivo* have provided valuable clues to guide the developments of next-generation airway transduction vectors and well-designed delivery approaches that use pharmacological interventions to overcome the extracellular and intracellular barriers to productive transduction.

### 3.1 Efficient airway transduction vectors

#### 3.1.1 Recombinant adenovirus vectors

rAd vectors were the first viral vectors tested in patients with CF (Zabner et al., 1993; Crystal et al., 1994). Although respiratory tract infections are the most common manifestation of adenovirus infection, partial correction of the Cl^−^ transport defect in nasal epithelium with CF using rAd-CFTR was only observed when the nasal epithelium was damaged during delivery (Joseph et al., 2001). This clinical finding was supported by the later discovery that the coxsackievirus and adenovirus receptor (CAR), which mediates the attachment and infection of adenoviruses type-2 and -5, is localized to the basolateral membrane of the human airway epithelium (Walters et al., 1999; Excoffon, 2020). Other disadvantages are that rAd-mediated CFTR expression in post-mitotic airway epithelial cells is transient and that rAd infection promotes strong cellular and humoral immune responses, which cause the destruction of transduced cell, as well as prevent repeated dosing (Harvey et al., 1999a; Harvey et al., 1999b). The anti-adenovirus immune responses might be further enhanced if the host has preexisting *Pseudomonas* infection (Tosi et al., 2004), which is a hallmark of *CF.*

#### 3.1.2 Helper-dependent adenovirus vectors

In attempts to solve the disadvantages of rAd for human gene therapy, helper-dependent adenovirus vector (HD-Ad) was developed (Brunetti-Pierri and Ng, 2006). The HD-Ad, in which all viral-encoded genes are removed, obviates the T-cell responses to cryptic viral protein expression; thus, it induces less inflammation and prolongs gene expression in the airways (Cao et al., 2005; Kushwah et al., 2008). It was reported that HD-Ad vectors were effective to express CFTR in the lungs of CF knockout mice (Koehler et al., 2003) and efficiently transduced large experimental animals like rabbits (Koehler et al., 2005) and ferrets (Yan et al., 2015a) *via* aerosol delivery. The large capacity of HD-Ad vectors (up to 37 kb) can be used to deliver both a gene editing endonuclease system and donor DNA for homologous recombination in a single vector (Xia et al., 2018; Zhou et al., 2019). It also enables the application of *piggyBac* transposase-mediated integration (Cooney et al., 2018b) or programmable nuclease-mediated targeted insertion (Xia et al., 2019; Bandara et al., 2021), which migrate the transgene cassette from the rAd genome to the chromosome, providing a solution to transient expression. Additionally, the application of sodium caprate (Gregory et al., 2003) or lysophosphatidylcholine (LPC) (Koehler et al., 2005) disrupts the tight junctions and allows for rAd vector to access the CAR at the basolateral cell surface to initiate transduction. Notably, the administration of HD-Ad vector formulated with LPC efficiently transduced airway basal cells in lungs of mice and pigs (Cao et al., 2018). Given that the airway basal cells are a multipotent progenitor for airway repair and regeneration (Hong et al., 2004; Hajj et al., 2007; Rock et al., 2009), integration of a *CFTR* expression cassette into basal cells allows the functional *CFTR* expression in their differentiated progeny.

#### 3.1.3 Recombinant adeno-associated virus vectors

When AAV-based CF gene therapy began in 1990s, rAAV2 was the only available serotype vector. Although the airways are not a natural host of AAV2, its application in lung gene transfer was rationalized by its wide tropism, and the results from preclinical studies demonstrated that rAAV2 was able to productively transduce the lungs of rabbits and rhesus macaques (Conrad et al., 1996; Beck et al., 1999; Beck et al., 2002; Fischer et al., 2003). However, later studies on rAAV2 transduction biology in the cell culture model of polarized human airway epithelium (HAE) at an airway-liquid interface (ALI) discovered that rAAV2 poorly transduced human epithelium from the apical membrane. The apical transduction of rAAV2 in HAE-ALI was 100-fold less efficient than that in the polarized cultures derived from the primary airway epithelial cells of rhesus macaques (Liu et al., 2007). Studies also found that, while transgene expression of rAAV2 apical infection was 200-fold lower than that of basolateral infection in HAE-ALI, a substantial amount of apically internalized rAAV2 genomes was detected (Duan et al., 1998). The lack of a correlation between vector entry and transgene expression suggested that the inefficiency was not primarily due to vector binding and endocytosis; rather, it was due to the failure to establish productive transduction. This observation was consistent with the outcome of clinical trials, specifically, the persistence of the tgAAVCF genomes but undetectable level of vector-derived *CFTR* mRNA in the recipients’ airways (Aitken et al., 2001). Subsequent research on vector intracellular trafficking revealed that impaired vector nuclear transport was the rate-limiting step in rAAV productive transduction in polarized airway epithelium (Duan et al., 2000). This post-entry block is responsive to the inhibition of proteasome activity, and it can be overcome by transient pharmacological intervention during or post transduction (Yan et al., 2002; Yan et al., 2004; Ding et al., 2005; Jennings et al., 2005; Monahan et al., 2010).

Another limitation of rAAV vectors in *CFTR* gene transfer is their relatively small package capacity (∼4.9 kb) (Dong et al., 1996; Flotte, 2000). The 4.5-kb *CFTR* coding sequence approaches the size of the AAV genome (4.68 kb); thus, tgAAVCF used the intrinsic promoter activity of the AAV2 inserted terminal repeat sequence (ITR) for *CFTR* expression (Flotte et al., 1993). Although studies in non-human primates supported this application, subsequent studies found that the cryptic promoter activity within AAV ITR was too weak in human airways and that the incorporation of an 83-bp synthesized sequence of essential transcriptional motifs (tg83 promoter) to tgAAVCF achieved a three-fold increase in the efficacy of CFTR functional expression in HAE-ALI (Zhang et al., 2004). To free up room for a stronger promoter, research for CFTR structure and function attempted to identify shortened *CFTR* minigene with only minimal reduction in its function (Carroll et al., 1995; Zhang et al., 1998; Sirninger et al., 2004; Cebotaru and Guggino, 2014). The most promising version is the CFTR∆R, whose size was reduced by 156 bp with a partial deletion (amino acid residues: 708–759) at the N-terminal portion of the R-domain. CFTR∆R generated similar Cl^−^ channel function *in vitro* and rescued the lethal intestinal phenotype in *CFTR* knockout mice *in vivo* (Ostedgaard et al., 2002). The use of *CFTR*∆R released enough room for the incorporation of another 100-bp enhancer element to strengthen the activity of the tg83 promoter. The resultant rAAV genome (AV2. F5tg83CFTR∆R) of 4.89 kb proved effective for the functional expression of CFTR in human CF airway epithelial cultures *in vitro* and transcription of *CFTR* mRNA in ferret airways *in vivo* (Yan et al., 2015b; Tang et al., 2020b).

The toolbox of rAAV-based vectors has largely expanded since 2000 (Asokan et al., 2012). Now, it is known that rAAV1 (a naturally occurring serotype) (Yan et al., 2006; Yan et al., 2013b) and rAAV2.5T (a variant selected from directed evaluation of an AAV capsid library in HAE-ALI) (Excoffon et al., 2009) are much more efficient than rAAV2 in apically transducing HAE-ALI. The post-entry block responsive for proteasome inhibition appears ubiquitous for parvovirus capsids: it was effective for most AAV serotypes and variants (Yan et al., 2002; Yan et al., 2006), as well as human bocavirus 1 (HBoV1) (Yan et al., 2013a). Composed of the human airway tropic AAV2.5T capsid and the most potent rAAV-CFTR construct, the next-generation rAAV-based *CFTR* transfer vector (AV2/2.5T.F5tg83CFTR∆R) was developed (Tang et al., 2020b). Currently, preclinical studies are being conducted by Spirovant Sciences, Inc. (Philadelphia, PA) to establish the efficacy of this new delivery system formulated with transduction augmenter (proteasome inhibitors) in the lungs of CF ferrets

#### 3.1.4 Lentiviral vectors

Lentiviral vectors derived from immunodeficiency viruses can transduce both dividing and non-dividing cells, and transgene expression from the integrated viral genome likely persists for the entire life cycle of recipient cells (Milone and O'Doherty, 2018). Although the insertional mutagenesis is an inherent safety concern, it has not yet been proven in clinical trials using lentiviral vectors (Schlimgen et al., 2016; Biasco et al., 2018). Because the lung airways are not a natural lentivirus host, lentiviral vectors used for airway transduction are necessitated to pseudotype with appropriate envelope proteins (Marquez Loza et al., 2019). As the hurdles to the success of non-viral strategies are poor transfection efficiency and transient expression, the UK CFGTC has since pursued a phase I/IIa lentiviral vector gene therapy trial for CF (Alton et al., 2017) with rSIV.F/HN-hCEF-CFTR (Mitomo et al., 2010; Griesenbach et al., 2012). rSIV.F/HN-hCEF-CFTR harboring the *CFTR* expression cassette from the clinically tested non-viral vector pGM169 is pseudotyped with F and HN (fusion and hemagglutinin-neuraminidase) proteins from the Sendai virus (SeV), which is a murine parainfluenza that causes mild disease in humans. A direct comparison between GL67A formulated pGM169 and rSIV.F/HN-hCEF-CFTR indicated that the lentiviral strategy was several log orders more efficient than the non-viral strategy in transducing airway epithelial cells (Alton et al., 2017).

#### 3.1.5 Airway transduction vectors derived from respiratory viruses

Despite the lungs having defense mechanisms against foreign invaders, respiratory viruses can circumvent these barriers to establish infection and replicate progeny (Nichols et al., 2008). Indeed, recombinant vectors from paramyxoviruses, such as human parainfluenza virus (PIV) (Zhang et al., 2005), respiratory syncytial virus (RSV) (Kwilas et al., 2010), and murine SeV (Ferrari et al., 2004), are able to efficiently transduce airway epithelium *in vitro* and in the lungs of mice *in vivo*. However, the vectors derived from these negative-sense, single-stranded RNA viruses only achieved transient expression, and their transduction induced strong adaptive immune responses. These disadvantages and the difficulties in vector production limit their path to clinical application.

DNA viruses associated with human respiratory disease include the nonenveloped double-stranded DNA (dsDNA) viruses from the family Adenoviridae and the newly discovered HBoV1, a single-stranded DNA virus from the family Parvoviridae (Allander et al., 2005). HBoV1 causes acute respiratory tract infection in young children (Kapoor et al., 2010; Peltola et al., 2013). The seroprevalence of HBoV1 capsid-specific immunoglobulin G is 13% and 59% in children and adults, respectively. However, seroconversion does not appear to prevent repeat infection by HBoV1 (Meriluoto et al., 2012). The full-length genome of HBoV1 has been cloned. While HEK293 cells are not permissive to HBoV1 infection, the transfection of the cloned HBoV1 duplex genome to these cells can produce infectious virions of HBoV1 (Huang et al., 2012). *In vitro*, HBoV1 efficiently infects polarized human HAE and produces progeny (Deng et al., 2013). As a relative of AAV, but an autonomous parvovirus, HBoV1 is easy to manipulate as a recombinant vector by the co-transfection of a rHBoV1 transfer plasmid and a helper plasmid, which provides viral components in trans, without the need for an adenovirus helper. rHBoV1 is able to efficiently transduce polarized HAE-ALI from the apical surface, but replication-competent rHBoV1 genomes would be expected in rHBoV1 viral stocks (Yan et al., 2013a). To eliminate this safety concern, a chimeric parvoviral vector, rAAV/HBoV1, was developed through parvovirus cross genera pseudopackaging. Composed of the clinically proven rAAV2 genome and the airway tropic HBoV1 capsid, rAAV2/HBoV1 maintains rHBoV1’s ability to efficiently transduce HAE-ALI from the apical membrane. Furthermore, the size of the HBoV1 genome is 5.5 kb; thus, the HBoV1 capsid can comfortably accommodate an oversized rAAV genome of up to 5.9 kb. With this 20% increased package capacity, rAAV2/HBoV1 vectors can use a strong promoter (and incorporate necessary transcriptional regulation elements) to express the full-length *CFTR* (Yan et al., 2013a). rAAV2/HBoV1 has demonstrated transduction in ferret lungs *in vivo*, enabling preclinical studies in CF ferret models (Yan et al., 2013a; Yan et al., 2017).

### 3.2 Suitable animal models in preclinical studies

CF animal models are essential in preclinical studies to validate the therapeutic strategy, to determine gene transfer agent dosing, administration routes, and application schedules in clinical trials. Soon after the discovery of *CFTR*, many CF mouse models were developed. However, because mice with CFTR mutations did not develop “spontaneous” chronic bacterial infection and/or inflammation in the lungs, CF mice are not a suitable animal model for testing gene therapies of CF lung disease (Wilke et al., 2011). To date, this problem has been solved by the developments of large-animal CF models (Yan et al., 2015a). *CFTR* knock-out (KO) ferrets (Sun et al., 2008) and pigs (Rogers et al., 2008) recapitulate many features of the lung disease phenotype observed in humans with *CF.* Lentivirus-, rAd- and rAAV-mediated *CFTR* lung gene transfer in *CFTR*-KO pigs have demonstrated the proof-of-concept for gene therapy in the hostile environment of CF lungs (Cooney et al., 2016; Steines et al., 2016; Cooney et al., 2018b). However, CF is a progressive disease, the lung disease phenotypes do not present at newborn and young age, in both ferret and pig models. For ferret, it usually takes a year or more to develop an infected CF lung. Both pig and ferret CFTR-KO models have severe gastrointestinal defects before and after birth, this makes them particularly difficult to rear beside of the low survival rate of the newborns. Thus, the utility of the first-generation CF ferrets and pigs as preclinical models for gene therapy has been limited (Yan et al., 2015a). The newly developed CFTR modulator-responsive CF models, e.g. *CFTR* ^G551D^ ferret (Sun et al., 2019), are even more suitable for preclinical studies with greatly increased survival rates and reduced difficulty in caring for sick animals. Administration of Ivacaftor (VX-770) during the gestation period protects the pancreas and intestine of the CF kits. After birth, *CFTR* ^G551D^ ferrets grow normally when Ivacaftor is given. Withdrawal of the drug reinitiates disease in the pancreas, gut, and lung at any age. Furthermore, these models express malfunction CFTR proteins, thus, immune responses to the transgene product, CFTR, are not expected to occur (Yan et al., 2019).

### 3.3 Compatibility of delivery systems and animal models in preclinical studies

Previous rAAV CF lung gene therapy clinical trials have highlighted the importance of the compatibility of delivery systems and animal models in preclinical studies. Before entering clinical trials, tgAAVCF was tested in rabbits and rhesus macaques with a single dose and repeat dosing. Vector-specific *CFTR* mRNA expression and the viral genome were detected in the macaques up to 180 days after infection (Conrad et al., 1996; Beck et al., 1999). These studies supported the utilities of AAV2 capsid for transduction and the AAV2 ITR promoter for transgene expression. However, the outcomes of *CFTR* expression from clinical trials were undesirable. Although it could be argued that the preclinical studies were not conducted in CF lungs, side-by-side direct comparison of the rAAV2 transductions in polarized human and monkey airway epithelial cultures revealed a large cross-species tropism difference of this AAV serotype capsid: The apical transduction of rAAV2 in the ALI cultures differentiated from primary airway cells of rhesus macaques was 100-fold more efficient than that in polarized HAE-ALI cultures (Liu et al., 2007). Similarly, a new AAV capsid variant (H22) identified from directed evolution of the AAV capsid library in the pig airway *in vivo* did not effectively transduce polarized HAE-ALI *in vitro*. Thus, despite the fact that AAVH22 vector, AAVH22/F5tg83CFTRΔR, effectively expressed *CFTR* in lungs of CF pigs to correct the defects of Cl^−^transport, pH of the airway surface fluid, and bacterial killing (Steines et al., 2016), its potential for application in humans is limited. Of note, rAAV2.5T and AAV/HBoV1 vectors are able to transduce ferret lungs *in vivo* (Yan et al., 2017; Tang et al., 2020b). This cross-species compatibility in vector tropisms enabled a more predictive preclinical path to evaluate the efficacy of gene therapy and to optimize vector delivery in the CF lungs of the *CFTR* ^G551D^ ferret.

## 4 Challenges

Early studies in humans carrying splice variants in *CFTR* (c.1393-1G>A [1525-1G>A], an exon 10 in-frame skipping) suggested that as little as 8% of the normal *CFTR* transcript was sufficient to preserve normal lung function (Chu et al., 1992; Chu et al., 1993). *In vitro* reconstitution experiments showed that polarized HAE cultures derived from mixtures of non-CF and ∆F508/∆F508 primary human airway epithelial cells at a 2:8 ratio (WT:CF) restored ∼70% of CFTR-mediated Cl^**−**^transport in 100% wild-type cultures (Farmen et al., 2005). Although these lines of evidence suggest that modest endogenous *CFTR* expression may suffice to improve CF lung disease, complementation of the defect in lung function by ectopic *CFTR* expression in CF airways is complicated because *CFTR* expression is highly regulated at different levels in the airways and in different epithelial cell types (Jiang and Engelhardt, 1998; Davis and Wypych, 2021). Additionally, the majority of cell types on the airway surfaces that are accessible to *CFTR* gene transfer vectors are terminally differentiated with a defined lifespan; thus, gene therapy necessitates repeat dosing to achieve unintermitted CFTR expression in the lungs with *CF.*

### 4.1 Heterogeneity in *CFTR*-expressing cell types in lungs and pathophysiologically relevant cell targets for gene therapy

**FIGURE 2:**
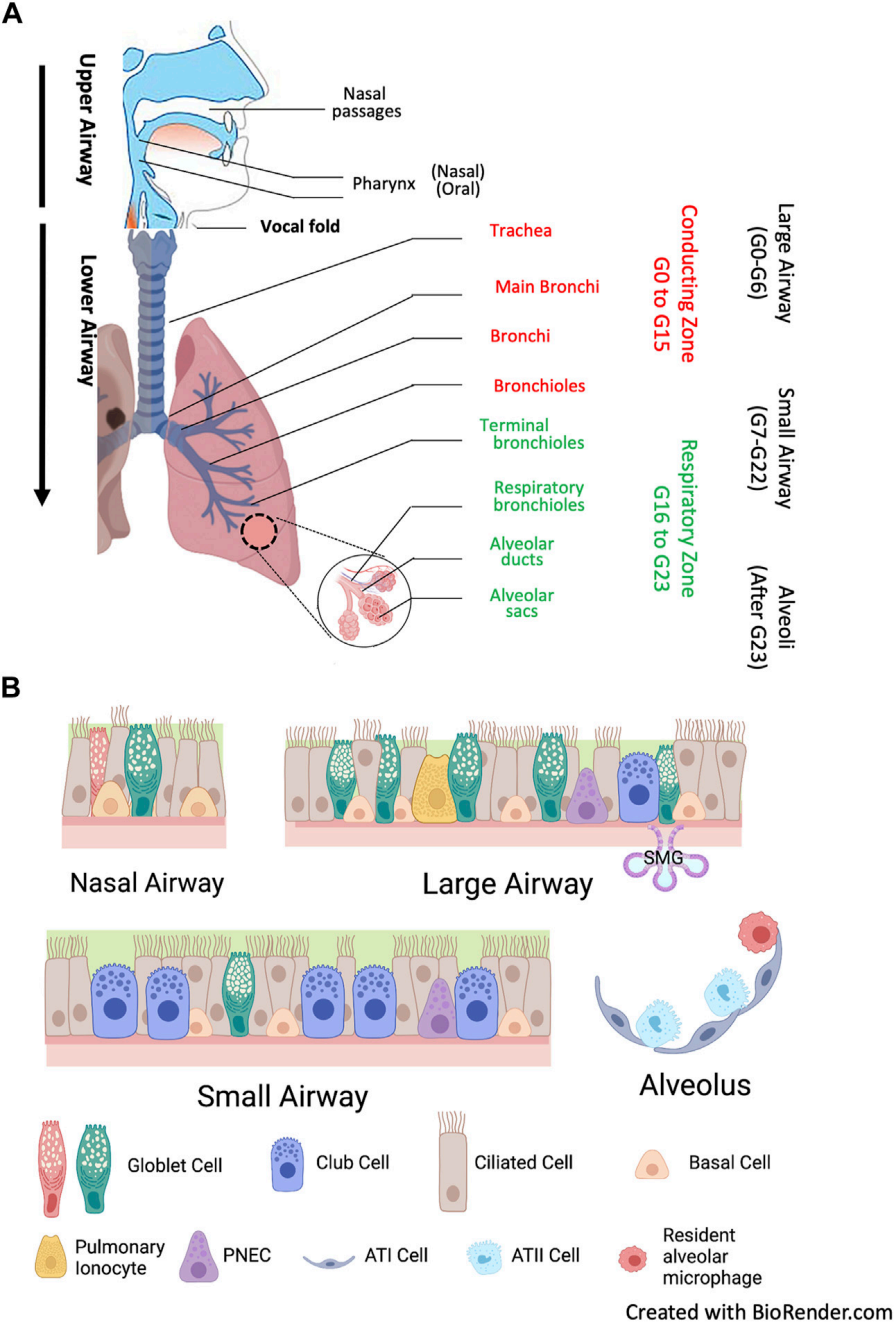
Human airways and major airway epithelial cell types. **(A)** Anatomy. The human airways are divided into the upper airways, lower airways and the lung parenchyma. The upper airways include the nose and nasal passages, paranasal sinuses, the pharynx, and the portion of the larynx above the vocal folds (cords). The lower airways include conducting zone from generation (G) G0 to G16, and respiratory zone from G17 and beyond. The trachea and proximal bronchi (generation G0 to G6) are considered as large airways. The small airways are distal bronchioles whose diameter is smaller than 2mm, starting from G7 the terminal bronchioles in conducting zone to the respiratory bronchioles in respiratory zone. After G23, the airway epithelium merges with the alveolar epithelium with pneumocyte type I and type II cells (alveolar type I and II cells, ATI and ATII cells) in lung parenchyma. **(B)** Airway epithelium is a continuous cellular layer with epithelial cell populations and functions vary along the respiratory tree. It is pseudostratified in the nasal and large airways, becoming columnar and cuboidal in the small airways. Submucosal glands (SMG) are majorly found in large airways. Major surface epithelial cell types are columnar ciliated cells, goblet cells, club cells and basal cells. Their composition is similar in large and small airways, but with gradually less ciliated cells, and the secretory cells shift from goblet cells to the dome-shaped club cells. Airway basal cells have the capacity to differentiate into the major cell types of the airways, and a subset of club cells can differentiate to ciliated cells and goblet cells. Pulmonary ionocytes express high level *CFTR*, but the mechanism by which they contributed to cystic fibrosis are still largely unknown. Ionocytes may be absent in the small airways. Pulmonary neuroendocrine cells (PNEC) serve as key communicators between the immune and nervous system.

The respiratory system is divided into the upper airways and lower airways. The conducting airways are defined as those sections of the respiratory tract which do not directly participate in gas exchange, and the distal airways include terminal and respiratory bronchioles and alveoli. The airway epithelium begins as a ciliated pseudostratified columnar epithelium in the trachea and slowly transition to that of a non-ciliated simple cuboidal epithelium in the terminal bronchioles. Human airways are lined by a variety of cell types, primarily including ciliated, mucous, and secretory cells on the surface layer, as well as the basal cells that are secured to the basement membrane (Figure 2). Each of these cell types plays a functionally distinct role and expresses different levels of *CFTR* (Davis and Wypych, 2021). Ciliated columnar cells are the dominant cell type throughout the surface epithelium of the large and small airways and thereby play a central role in the first line of lung defense and a pivotal role in airway homeostasis through MCC. Their amount increases with airway branching, from 47% ± 2% in the trachea to 73% ± 1% in the small airway epithelium (Raman et al., 2009). Goblet cells are the primary secretory cells in proximal cartilaginous airways, which also contain numerous submucosal glands (SMG) (Widdicombe and Wine, 2015). SMG consist of acini and tubules connected to the surface epithelium *via* collecting and ciliated ducts and house diverse cell types: serous, secretory, goblet, myoepithelial and ciliated cells, as well as basal cells. The terminal bronchioles contain basal cells, secretory club cells, ciliated cells, and neuroendocrine cells, whereas the respiratory bronchioles contain primarily club cells. Alveoli represent the most distal portion of the respiratory tract, where the major cell types are type I and type II pneumocytes and alveolar macrophages, which are the most abundant innate immune cells in the distal lung parenchyma (Whitsett et al., 2019).

CFTR protein and mRNA expression are abundant in type II pneumocytes (alveolar type II cells, ATII) in the lung parenchyma, club cells in the respiratory bronchioles, and serous cells of SMG (Engelhardt et al., 1992; McCray et al., 1992; Engelhardt et al., 1994). However, *CFTR* is only expressed in a small subset of cells in the surface airway epithelium. Recent research using single-cell RNA sequencing (scRNA-seq) has uncovered more cellular heterogeneity within the airways and the correlations with *CFTR* expression (Montoro et al., 2018; Plasschaert et al., 2018). The newly identified pulmonary ionocytes express *CFTR* at the highest level per cell; thus, they represent a relatively large proportion of the total *CFTR* transcripts in the airways. However, ionocytes comprise <1% of epithelial cells along the conducting airways, even though they are abundant in the submucosal gland ducts and the surface airway surrounding these glands (Yu et al., 2022). Ionocytes are involved in pH regulation, ion transport, and hydration of the airways, however, it remains unclear whether ionocyte depletion in human airways leads to chronic inflammation and spontaneous bacterial infection, which are the hallmarks of *CF.* In mice, *FOXI1* gene is necessary for the expression of ionocyte markers in the trachea. Knockout of *FOXI1* in mouse caused a loss of *CFTR* expression and disrupts airway fluid and mucus physiology (Montoro et al., 2018). However, there are differences in airway epithelial cell biology between mice and humans (Pan et al., 2019). Patients with homozygous missense mutations in *FOXI1* present with early-onset sensorineural deafness and distal renal tubular acidosis, but not associate with lung disease (Enerback et al., 2018). As ionocytes are not the sole *CFTR*-expressing airway epithelial cell type, targeting ionocytes in the treatment of the lungs with CF in humans may not be crucially necessary (Barbry et al., 2021).

Analyses from scRNA-seq combining cell number and level of *CFTR* expression suggested that a rank order for total *CFTR* expression in human superficial airway epithelium was secretory cells, basal cells, ionocytes, and ciliated cells, respectively (Okuda et al., 2021). Several scRNA-seq studies have drawn a consistent picture with secretory cells as major sites for *CFTR* expression in human surface airways (Deprez et al., 2020; Goldfarbmuren et al., 2020; Habermann et al., 2020; Miller et al., 2020). Due to the rarity of ionocytes, it is possible that the proportion of *CFTR* signals transcribed from other cell types that express lower levels of *CFTR* but are abundant in the surface airways may better correlate with CFTR protein function required for airway surface fluid homeostasis. Indeed, a recent study suggested that secretory cells dominate CFTR expression and function in human airway epithelia, and that CFTR-mediated hydration is associated with secretory cells (Okuda et al., 2021). Thus, while CF therapy may need to restore CFTR function in multiple cell types, including ionocytes, a major target likely is the airway secretory cells. However, secretory cells in the conducting airways (goblet cells) also differ in their biology to those in the bronchioles (club cells) (Dean and Snelgrove, 2018); thus, CFTR may play a different role in these two types of secretory cell. Additionally, it is difficult for gene transfer agents to access the secretory cells underneath the surface airways, such as in areas surrounding the SMG.

Ciliated cells are the first contact accessible to the gene transfer agents delivered from the airway lumen. They had long been thought to be a target for CF gene therapy (Kreda et al., 2005; Zhang et al., 2009). However, more recent studies by scRNA-seq discovered that little to no *CFTR* mRNA transcripts are detected in this cell type. In light of this, it remains unclear whether ectopic expression of *CFTR* in ciliated cells are able to compensate the CF lung function if *CFTR* expression is not sufficiently reinstalled in secretory cells and inonocytes. It is also unknown whether the vector infection and the exogenous *CFTR* expression in this cell type will cause ciliopathies that impact its functions in MCC and normal innate immunity. Comprehensive preclinical studies of *CFTR* delivery in animal models of CF and an understanding of CFTR function at the cellular level to control airway clearance and innate immunity will help to resolve these unanswered questions.

### 4.2 Complexity of CF pathogenesis

CF is a multi-organ disease, and pulmonary disease is the main cause of morbidity and mortality. While CFTR is commonly known an ion channel protein that conducts chloride (Cl^−^) and bicarbonate (HCO_3_^−^) transport on the apical membrane of the epithelial cells, the pathophysiology of CF is more challenging than a mere dysregulation of epithelial ion transport. CFTR also interacts with other ion channels, such as epithelial sodium channel (ENaC) (Shei et al., 2018), to regulate the airway surface fluid (ASL) movement, pH homeostasis and mucus viscosity (Wu et al., 2018). Therefore, this interaction plays a major role in the MCC and innate immunity to defend the lung infections. The loss of functional CFTR in CF results in dysregulation of their functions. In the lung airways, the *CFTR* gene defect results in the loss of a functional CFTR expression (either absence or malfunction of CFTR protein). The abnormal ion conductance leads to dehydration of ASL, impaired MCC, airway obstruction by viscous sputum and abnormally thick and sticky mucus promotes chronic infection and inflammation. The consequence of this cascade is inflammation and progressive chronic endobronchial bacterial infection, the hallmarks of CF lungs, resulting in permanent lung damages (Ratjen, 2009; Huang et al., 2021). Additionally, CFTR is also expressed by immune cells, including alveolar macrophages and neutrophils, which help maintaining immunological and physiological homeostasis in the lungs and are the front line of cellular defense against pathogens that were not eliminated by the mechanical defenses of the airways (Yoshimura et al., 1991). Impaired immune cellular defense has been reported in CF (Leveque et al., 2017). While the current gene therapy development efforts target life-threatening lung disease *via* CFTR replacement (gene addition) or mutation repair (gene editing), it is worth mentioning that Cl^−^ secretion in airway epithelial cells is not limited to CFTR. *CFTR* knockout mice do not develop the typical lung disease phenotype seen in humans with CF, which is probably at least partially attributable to up-regulation of the alternative chloride channels (Guilbault et al., 2007). CFTR has also been proposed to inhibit ENaC activity through direct physical interactions, and dehydration of CF ASL is driven by ENaC (Stutts et al., 1995). Thus, stimulation of alternative chloride channels (Xu et al., 2005; Liu et al., 2015; Quesada and Dutzler, 2020) or inhibition of sodium absorption (Blacona et al., 2022) may be able to compensate for the lack of CFTR-mediate Cl^−^ secretion in CF airways or to maximize the efficacy of gene therapies aiming at *CFTR*. Notably, it was reported that proteasome modulation agent doxorubicin facilitated long-term functional inhibition of ENaC currents. Doxorubicin has been used as an augmenter to enhance rAAV-mediated *CFTR* transfer in human airway epithelium and it was found that the inhibition of ENaC activity was predominantly attributed to a doxorubicin-dependent decrease in *γ*-ENaC subunit mRNA expression and an increase in *γ-ENaC* promoter methylation, independent of CFTR vector administration (Zhang et al., 2004).

### 4.3 Repeat dosing

Ciliated cells, goblet cells, and club secretory cells on surface airway epithelia are the primary cell types accessible to the *CFTR* transfer vectors applied luminally. These cell types are terminally differentiated with a defined lifespan, except for a subset of club cells in small airways which may have the ability to differentiate into other cell types (Rawlins et al., 2009; Dean and Snelgrove, 2018). Even if basal cells, the stem cell progenitors underneath the surface layer, could be exposed to the vectors by transient disruption of the tight junctions, the episomal rAAV genomes in the transduced cells will be diluted out on serial passage during the cell proliferation. Because of the relatively slow turnover rate of epithelia (Rawlins and Hogan, 2008), treatment of CF patients can be periodic as durable, functional complementation in the transduced cells likely persists for a certain period or through their lifespan. Nevertheless, using rAAV for the treatments to CF lungs requires multiple repeated dosing to achieve sustained CFTR expression for the life of the individual. This notion is likely also true for the integrating lentiviral vectors, unless the basal cells are transduced.

Pre-existing humoral immunity to AAV capsids is a potential barrier to rAAV vector-mediated gene therapy, especially following re-administration of the same rAAV vector (Boutin et al., 2010; Masat et al., 2013; Mingozzi and High, 2013). Besides the elicitation of neutralizing antibodies (NAbs), vector immunogenicity also represents the major cause of the destruction of transduced cells, which is mediated by the induced cytotoxic T lymphocytes (CTL) response after transduction (Vandamme et al., 2017; Ronzitti et al., 2020; Verdera et al., 2020). While little is known of the effects of T cells on the duration of rAAV transduction administrated through airways, the involvement of CD^8+^ T cells in the destruction of rAAV-transduced hepatocytes was reported in the rAAV2 hemophilia B clinical trial (Mingozzi et al., 2007). Transgene protein product-specific CTL was also observed in rAAV trials for Duchenne’s Muscular Dystrophy (Mendell et al., 2010) and α1-antitrypsin (Calcedo et al., 2017). Lentiviral vectors may face similar or the same immune response problems as rAAV vectors.

Pharmaceutical immunosuppression is likely able to dampen the immune response to repeat administration of rAAV, through global effects, T-Cell or/and B-Cell specific effects, or other mechanisms (Jiang et al., 2006; Arruda et al., 2009; Mingozzi et al., 2013; Chu and Ng, 2021). However, the approaches that had been tested in gene therapy trials *via* local (intramuscular injection) or systemic (intravenous injection) rAAV delivery necessitate patients to be maintained with daily administration of a regime of immunosuppressants for a relative long period (Corti et al., 2014; Corti et al., 2015). Such treatment might result in chronic immune suppression that is intolerable in CF patients, as the CF lungs are prone to bacterial infections. A recent study of repeat dosing with rAAV1 in the lungs of rhesus macaques demonstrated that a brief course of treatment with methylprednisolone succinate prior to infection boosted the efficiency of transduction and increased the longevity of transgene expression (Yanda et al., 2022). Methylprednisolone is a mild immunosuppressant, it has been used in patients with CF for several indications, including bronchiolitis, bronchial hyperreactions, aspergillosis, and mild-to-moderate obstructive pulmonary disease (Lai et al., 2000; Thomson et al., 2006). However, as the CF condition is not duplicated in non-human primates, the application of methylprednisolone for rAAV repeated dosing requires studies using CF animal models (pigs or ferrets) to establish the minimal effective dose and to clarify the safety and feasibility.

## 5 Gene editing

Theoretically, genome manipulation is able to precisely correct (edit) any mutations of a target gene *via* homologous recombination (HR) without altering its endogenous patterns of expression. Although the efficiency of HR in mammalian cells is extremely low, it was found that induction of double strand DNA break (DSB) could raise the incidence of HR by multiple orders of magnitude. Thus, a key step to genome manipulation or gene editing is the generation of a sequence-specific DSB at the target gene. Thus, gene editing requires sequence-specific endonucleases to create DSB at desired site and relies on the followed repair of the DSB by two cellular DNA damage repair mechanisms: the non-homologous end joining (NHEJ) and the homology-directed repair (HDR) (Ceccaldi et al., 2016). However, the gene therapy field long lacked such a tool of “molecular scissors” until the development of engineered programable nucleases in 2010. Zinc finger nucleases (ZFN) (Urnov et al., 2010) and transcription activator-like effector nucleases (TALEN) (Zhang et al., 2011) are the first two programable nucleases developed to manipulate a gene of interest with practically high specificity and efficiency. ZFNs are fusion proteins built with an array of zinc finger domains, that each recognizes a 3 bp DNA motif, attached to the endonuclease domain of the bacterial FokI restriction enzyme. TALENs are also proteins fused to the catalytic domain of the FokI nuclease. They use transcription activator-like effectors (TALEs) rather than zinc fingers to recognize specific DNA sequences. ZFN- and TALEN -mediated HDR to correct the mutations at endogenous *CFTR* loci in CF patient-derived iPSCs (Crane et al., 2015; Fleischer et al., 2020) and TALEN-mediated site-specific integration of a CFTR minigene at the AAVS1 safe harbor (Xia et al., 2019) are the examples of using these tools in the research for CF gene therapy.

The later application of the bacterial “adaptive immune system”, namely the clustered regularly interspaced short palindromic repeats (CRISPR) system, in mammalian genetic engineering has exhibited a facile, highly versatile and efficient gene editing tool that the field of gene therapy has long desired (Cox et al., 2015; Araldi et al., 2020). Distinct from the ZFNs and TALENs that require custom protein design for each target, CRISPR-associated proteins (Cas) are enzymes that associate with CRISPR RNAs to bind to and alter DNA or RNA target sequences. The CRISPR system for gene editing is comprised of a Cas and a single guide RNA (sgRNA) that site-specifically guides the complex to the gene of interest. Acting as a programmable endonuclease (with Cas) (Jinek et al., 2012) or nickase (with nCas) (Ran et al., 2013), the complex is able to generate a DSB or create a single-stranded break in the targeted gene in a site-specific manner. When catalytically inactive Cas (dCas) is used, the complex binds to the target without creating strand breaks (Liu et al., 2016). CRISPR-based gene therapy has shown promise in sickle cell disease by correcting the mutation in hematopoietic stem cells *ex vivo* (Frangoul et al., 2021) and *in vivo* in Leber congenital amaurosis (LCA) by deleting delete the dominantly inherited intronic IVS26 loss-of-function mutation (Maeder et al., 2019) and in transthyretin amyloidosis by disrupting the disease-causing allele (Gillmore et al., 2021). It presents new opportunities to advance CF gene therapy from conventional gene addition to precise gene correction of any *CFTR* mutation at the endogenous locus, by which, theoretically, the complexities of CFTR regulation at the cellular level in the lung could be solved (Yan et al., 2019; Vu and McCray, 2020).

### 5.1 CRISPR-based gene editing

The CRISPR/Cas-nuclease approach relies on the generation of a DSB at the desired site of a target gene. The DSB activates the cellular DNA damage response (DDR) by the recruitment of repair factors to the site of DNA damage, and thus, gene correction is enabled by the cellular DSB repair machinery, either through homology-directed repair (HDR) or non-homologous end joining (NHEJ) (Lieber, 2010). With the provided template for gene correction, the HDR response enables homologous recombination (HR) between the template and the target gene at the cleaved site to introduce desired correction, insertion, or deletion (Paquet et al., 2016). NHEJ directly ligates the ends of the breaks, however, it can generate unpredictable insertions and deletions of various lengths (indels) (Betermier et al., 2014). HDR and NHEJ are competing processes. Under most conditions, NHEJ is more efficient than HDR. HDR is highly cell cycle-dependent; it occurs only in the G2 and S phases of the replication cycle (Heyer et al., 2010). Thus, most mitotically quiescent cells and well-differentiated cells do not support HDR. Although CRISPR/Cas-nuclease generates DSBs in a site-specific manner, unintended off-target cleavage is a safety concern (Fu et al., 2013). Moreover, unpredictable rearrangement and translocation, as well as a high ratio of undesired indel byproducts, are potential results of the repair (Jeggo, 1998; Kosicki et al., 2018).

**FIGURE 3:**
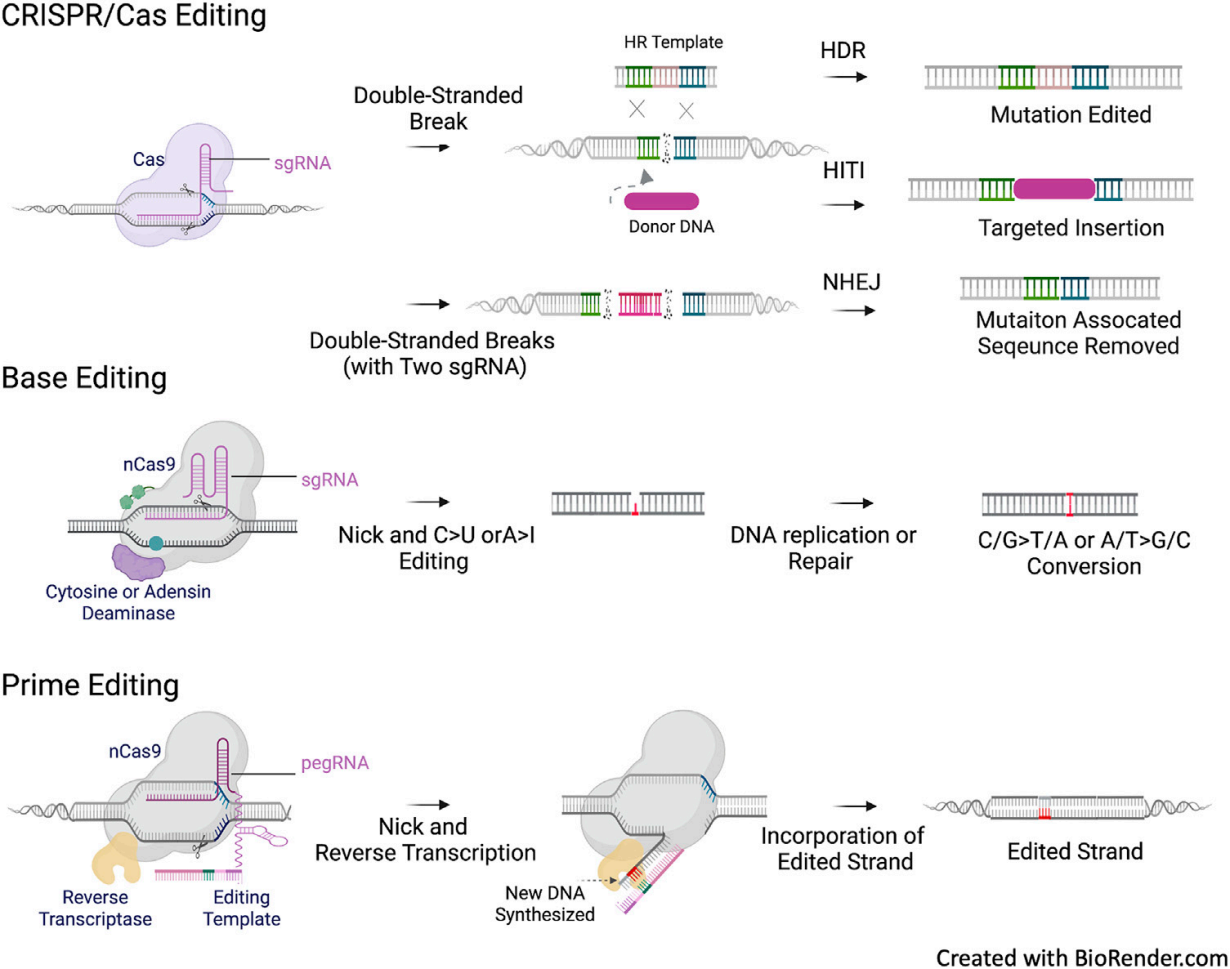
*CFTR* mutation types and CREIPSR-based Gene Editing. CRIPSR/Cas editing approaches rely on the generation of double stranded DNA break (DSB) and the following repair *via* non-homologous end joining (NHEJ) or homologous recombination (HR). NHEJ can be used for the allele specific repair of splicing mutation in the intronic sequence, such as the 3272-26A>G (c.3140–26A>G) and 3,849 + 10kbC>T (c.3718–2477C>T) CFTR mutations. Homology-independent targeted insertion (HITI) is an approach based on NHEJ. Theoretically, HITI can universally address all *CFTR* variants independent of genotypes, the donor DNA can be a splicing acceptor associated CFTR cDNA sequence to be inserted into the intronic sequence of *CFTR* to reconstitute a mutation free mini gene, or a CFTR expression cassette to be inserted into the safe harbor of the chromosome, such as AAVS1. Homology-directed repair (HDR) requires a template for HR to correct point mutation or small deletion/deletion. Base editing and prime editing do not require the generation of DSB and DNA template for correction. Base editing is only used to correct single base point mutation, and primer editing can be used to correct point mutation as well as short deletion/insertion. Same as HDR, the correction options of base editing and prime editing are mutation dependent.

Base editing (BE) (Gaudelli et al., 2017) and prime editing (PE) (Anzalone et al., 2019) are the next generation of CRISPR-based gene editing without the need for DSB. BE utilizes a dCas9 or nCas9 fused with a deaminase domain to edit specific loci, allowing for the introduction of point mutations through direct enzymatic C>T base conversion (cytidine base editor) or A>G base conversion (adenine base editor). However, the application of BE is limited by the presence of a compatible protospacer adjacent motif (PAM) at the mutation to be edited and by the availability of A>G and C>T transitions and C>G transversions (Rees and Liu, 2018). PE enables single-base substitution for all 12 potential transition and transversion reactions. Moreover, it facilitates alterations in short insertions or deletions without the need for a donor DNA template. PE consists of a Cas9 nickase fused with a modified reverse transcriptase and a multifunctional prime editing guide RNA (pegRNA), which directs the fusion complex to the target site for flexible PAM recognition and also encodes an RNA template for editing as a contiguous extension of the gRNA. These CRISPR-based gene editing tools are outlined in Figure 3.

### 5.2 Proof-of-concept gene editing of *CFTR* mutants

CRISPR-based gene editing approaches have been explored to correct certain *CFTR* mutations in cultured airway basal cells, iPS cells, and advanced cellular models, such as organoid cultures derived from patients with CF (Ensinck et al., 2021; Lee et al., 2021). HDR-based CFTR corrections have demonstrated the capacities to correct missense or nonsense mutations in exons, to ablate splice mutations, and to insert “super exons” from the DNA donor template provided along with the CRISPR components (Schwank et al., 2013; Firth et al., 2015). HDR has succeeded in repairing F508del *CFTR* in primary human airway basal cell cultures (Suzuki et al., 2020; Vaidyanathan et al., 2020) and G551D *CFTR* in proliferating ferret airway basal cells, to which the components of the CRISPR system were delivered by either transfection or rAAV infection. However, the efficiency of HDR is low because the error-prone NHEJ dominates the cellular DNA repair machinery. Next-generation sequencing analyses of the targeted amplicon from rAAV-infected ferret airway basal cells (without selection or enrichment) revealed a high fidelity with HDR of only 0.55% corrected G551 alleles associated with additional modifications, but the 13.21% correction rate of the G551D mutation with HDR (including imperfect HDR events) was lower than the 22.57% rate of undesired mutagenesis caused by NHEJ (Yan et al., 2022). Because NHEJ is efficient in both dividing and non-dividing cells, NHEJ-based gene editing, which only requires the expression of a Cas protein with pairs of gRNAs, appears practicable, particularly in the repair of aberrant *CFTR* mRNA transcripts caused by intronic splicing mutations. The efficacy of this gene-correction strategy has been verified in intestinal organoids and airway epithelial cells derived from patients with CF, who carry the mutations of c.1679 + 1.6kbA>G (1811 + 1.6kbA>G), or c.3140–26A>G (3272-26A>G) or c.3717 + 12191C>T (3,849 + 10kbC>T), with an allele-specific repair efficiency of ∼40% and complete functional recovery of the CFTR channel (Sanz et al., 2017; Maule et al., 2019). A more complicated NHEJ editing strategy is homology-independent targeted insertion (HITI) (Suzuki et al., 2016). HITI requires a donor DNA and targeted integration with correct orientation. It has been reported that targeted insertion of a splicing acceptor-associated *CFTR* minigene of exons 9–27 into *CFTR* intron eight of F508del/F508del airway basal cells was able to reconstitute the functional *CFTR* expression in the edited cells, although the efficiency was low. Other approaches include targeted insertion of a CFTR minigene expression cassette into a genome safe-harbor or the CFTR locus (Xia et al., 2019; Zhou et al., 2019).

BE is an attractive approach for gene editing based CF gene therapy because many disease-causing *CFTR* variants can be corrected using single base conversion (Geurts et al., 2020). Adenine base editor has been used to correct premature stop mutations in R553X and W1282X, as well as the 3,849 + 10 kb C>T splicing mutation in human airway epithelial cells at 38%–82% efficiency with minimal bystander edits and indels (Krishnamurthy et al., 2021). The repair of the *CFTR*-R785X mutation has been compared between BE and PE approaches in patient-derived intestinal organoids (Geurts et al., 2021). Adenine base editor demonstrated a correction efficiency of 9.1%, whereas the best-performing PE achieved a correction efficiency of 5.7%. Although both approaches outperformed the conventional HDR, which demonstrated a correction efficiency of 1.22%, BE appears to be superior to prime editing in terms of both safety and efficiency when the mutation is targetable. Nevertheless, PE can theoretically correct mutations that cannot be corrected with BE. PE has demonstrated its capacity to repair the most common *CFTR* mutation (F508del) in patient-derived intestinal organoids, although the efficiency was lower than that of HDR (Geurts et al., 2021).

### 5.3 *Limitation of the implementation of CRISPR/Cas* for *ex-vivo and in-vivo* lung gene editing

The applications of gene editing in humans are undergoing with several clinical trials: one is an *ex vivo* mutation repair strategy, using autologous CRISPR-Cas9 modified hematopoietic stem cells to treat hematopoietic stem cells (NCT04208529); another two are *in vivo* strategies for LCA, using rAAV to express spCas9 and two gRNAs in eyes (NCT03872479), and for transthyretin amyloidosis [(hereditary transthyretin amyloidosis with polyneuropathy (ATTRv-PN) and transthyretin amyloidosis-related cardiomyopathy (ATTR-CM)), using lipid nanoparticle (LNP) encapsulating Cas9 mRNA and sgRNA (NCT04601051). While these trials are promising, the implementation of CRISPR-based editing to many inherited diseases with more complex etiologies, like CF, will require more challenging approaches.

CRISPR/Cas9 HDR approaches can be used to target *CFTR* gene correction to airway stem cells. Currently, the *CFTR* gene editing approaches have been explored in cultured primary airway basal cells, iPS cells, and organoid cultures derived from patients with *CF.* Theoretically, gene editing in airway basal cells *in vitro* can be used for autologous cell therapy to regenerate airways with CFTR functional cells. It requires autologous corrected cells to generate a sufficient cell quantity through *ex vivo* expansion and to have the capacity to engraft, proliferate, and persist long-term in recipient airways without complications (Berical et al., 2019; King et al., 2020). However, unlike the current cell-based therapies such as the hematopoietic cell transplantation, an practical strategy to deliver autologous gene-edited airway basal cells for CF and any lung disease has not yet developed. As well, there is a lack of method to control the mucus and infection to allow engraftment of cells. An engraftment strategy would involve minimal airway injure, but the prospect of deliberately injuring the lungs of CF recipients to ablate the endogenous cells is daunting. (Berical et al., 2019).

Notably, for *in vivo* application, HDR is inefficient in the repair of DSBs in quiescent and differentiated cell types of the airway epithelium. The HITI approaches (Suzuki et al., 2016; Xia et al., 2019; Suzuki et al., 2020) appear compatible in editing basal cells and post-mitotic airway epithelia, however, a lifelong cure is only achievable if the airway basal cells are efficiently targeted for permanent correction. Editing of *CFTR* in vector-accessible surface airway epithelial cell types may produce durable CFTR activity for a certain period, but similar to the gene addition approaches, repeat dosing is required. Because *in vivo* lung gene editing encounters the similar dilemma in delivery as do the gene addition approaches and also the efficiency of current HITI efficiency to reconstitute a function expression of CFTR *in vitro* is still sufficiently low, studies have not yet progressed toward *CFTR* correction *in vivo* due to the lack of robust delivery methods to efficiently target basal cells. The current available viral vector systems have limited utility to deliver all the components of the CRISPR-based gene editing system with in a single vector, especially for the base- and prime editors that are large size cas9-based fusion proteins, thus, engineered DNA-free virus-like particles that can be used for *in vivo* editing may offer additional therapeutic options (Banskota et al., 2022). Although it is more challenging to edit basal progenitor cells *in vivo* than proliferating cells *in vitro*, the development of a robust delivery system enabling efficient basal cell targeting and correction will translate current proof-of-concept studies into real therapies for patients with *CF.*

## 6 Summary and prospect

Restoration of lung function in patients with CF relies on rebuilding normal homeostatic mechanisms that require the interaction between transgene-expressed CFTR and other ion channels to regulate effective airway clearance and innate immunity (Shah et al., 2016). Effective gene therapies for CF lung disease have been pursued since the discovery of *CFTR* in 1989. Researchers in both academia and industry have contributed significant resources and talent to tackling the challenges encountered in the long journey toward a cure for *CF.* Currently, gene editing is still in its infancy. rAAV based CFTR-replacement strategies for CF remain the primary hope for clinical success. Although now the field of CF gene therapy is equipped with the next generation rAAV based transduction vectors highly tropic to human airway, the greatest challenge is to deliver a vector to as many of the epithelial cells that line the surface airway as possible, and permanent correction requires the airway basal progenitor cells to be targeted. There is also an urgent need of safe and effective strategies to reduce the vector immunogenicity for repeated dosing. The safety is particularly important for the application of immunomodulation in CF lungs, which are prone to bacterial infection. While the discussion of this review is mostly focused on the applications of viral vector strategies, it is worth to note here that development of the polymeric nanoparticles as new delivery tool for gene therapy. A recent study showed that intratracheal delivery of nanoparticles-formulated thymulin-expressing plasmids was capable to penetrate the airway mucus barrier, which therapeutically reversed the key pathology of experimental allergic asthma in mice (da Silva et al., 2020). The nanoparticles-encapsulating mRNA delivery techniques are getting matured now, its application expand in the field of gene therapy rapidly. Currently, there is an ongoing CF clinical trial in phase1/2 to test the delivery of a drug called MRT5005, which uses lipid nanoparticles to deliver an mRNA encoding the full-length *CFTR*, to the CF lungs *via* nebulizer (NTC03375047; https://www.cff.org/Trials/Pipeline/details/10157/MRT5005). Thus, LPN-mediated Cas9 mRNA delivery for *in vivo* lung gene editing could become possible. The availability of large-animal CF models enables preclinical studies to validate new therapies, and also facilitates the basic biomedicine research to identify the pathophysiologically relevant target cell types for effective CF gene therapy and to test the new delivery tools in CF airways. CF is a complex disease, and the success of gene therapy for CF will hopefully also define a path for the treatment of other complex and devastating diseases.
